# Priority of spontaneous gender categorization of same-sex faces in young adults

**DOI:** 10.1093/scan/nsaf033

**Published:** 2025-04-22

**Authors:** Huang Zheng, Shihui Han

**Affiliations:** School of Psychological and Cognitive Sciences, PKU-IDG/McGovern Institute for Brain Research, Beijing Key Laboratory of Behavior and Mental Health, Peking University, Beijing 100081, China; School of Psychological and Cognitive Sciences, PKU-IDG/McGovern Institute for Brain Research, Beijing Key Laboratory of Behavior and Mental Health, Peking University, Beijing 100080, China

**Keywords:** EEG, face, gender categorization, P2 amplitude, sex

## Abstract

To cluster others into male and female groups during face perception is pivotal for appropriate social behaviours. What remains unclear is whether gender categorization of faces is mediated by the same pattern of cognitive and neural processes in women and men. The perception bias hypothesis predicts earlier gender categorization of female (vs. male) faces regardless of an observer’s gender. In contrast, the social task demand hypothesis predicts earlier gender categorization of faces that are of the same (vs. different) sex of an observer. We tested these predictions by recording electroencephalography signals to faces of one gender presented in a repetition condition and to both female and male faces in an alternating condition. The neural processes underlying gender categorization were assessed by quantifying repetition suppression of brain activities to faces in the repetition relative to alternating conditions. We found significant repetition suppression of a positive frontal-central activity at 170–210 ms after face onset (the P2 component) to female (but not to male) faces in women. However, repetition suppression of the P2 amplitude occurred to male (but not to female) faces in men. Our findings suggest that observers’ genders are pivotal for prioritization of gender categorization of male or female faces in young adults.

## Introduction

People undertake different forms of interactions with own-gender and other-gender individuals since early childhood. Classification of others by gender occurs spontaneously and implicitly duringface perception in both children and adults (Bennett et al. 2000, Quinn et al. 2002; Ito and Urland 2003; Miller et al. 2020). However, recent research has shown that gender categorization does not occur spontaneously to all perceived faces. Gender categorization gives priority to same-race over other-race faces and this priority is modulated by social task demands (Zhang et al. 2023). This finding raises an open issue regarding gender categorization, that is, whether women and men employ the same pattern of cognitive and neural processes during gender categorization of same-sex and different-sex faces. To address this question is critical for understanding gender differences in social interactions and communications that have been observed in both healthy and clinical populations (e.g. Wood‐Downie et al. 2021). The present study sought to clarify this issue by recording electroencephalography (EEG) to female and male faces in women and men.

Social categorization of faces depends on both low-level perceptual processes and high-level social task demands (Freeman and Johnson 2016, Zhang et al. 2023). Two different predictions regarding prioritization of gender categorization of same-sex or different-sex faces can be derived from previous studies of gender-related processing. The perception bias hypothesis posits that, for the mental processes that generate a gender percept, the ‘female’ relative to ‘male’ is positively coded by the presence of additional perceptual features or properties (Gandolfo and Downing 2020). For example, female faces perceived in everyday life have shared additional information such as hair or makeup, whereas male faces lack such shared information. This hypothesis has been used to interpret the response bias in reporting faces or body shapes as ‘male’ under conditions of uncertainty or ambiguity (Davidenko 2007; Armann and Bülthoff 2012, Watson et al. 2016, Gandolfo and Downing 2020). According to this hypothesis, additional perceptual features shared by different female faces may be used to classify these faces spontaneously and lead to a priority (earlier or easier) of gender categorization of female over male faces regardless of observers’ gender.

In contrast, the social task demand hypothesis assumes that social categorization of faces gives priority to those of higher relevance with social task demands in daily life situations along a specific dimension of facial properties (e.g. race or gender, Zhang et al. 2023). For instance, categorization of faces by race gave a priority to other-race over same-race faces because to quickly classify other-race faces as members of an outgroup would speed social decision-making during interracial interactions (Zhou et al. 2020). Gender categorization of faces helps to govern cross-sex interactions including romantic and mating partner selection. Such social tasks require observers to process different-sex persons individually but to classify same-sex persons as a group of competitors during mating partner selection (Darwin 1871), predicting a priority of gender categorization of same-sex over different-sex faces as an accustomed cognitive strategy of gender-related processing of faces. Such a cognitive strategy helps people to take similar actions in response to same-sex persons collectively as mating competitors, and in contrast, to treat different-sex persons individually for evaluation as a potential mating partner. Therefore, one may predict that women, in particular, young women, would show a priority of gender categorization of female over male faces and men, in particular, young men, would show a priority of gender categorization of male over female faces.

To test the discrepant predictions regarding the priority of gender categorization of faces in women and men, we recorded EEG to female and male faces in a repetition suppression (RS) paradigm developed in our recent studies of social categorization of faces (Zhou et al. 2020, Zhang and Han 2021, Zhang et al. 2023). In this paradigm, participants viewed different individuals’ faces of only one gender (female or male) category in a repetition condition or different individuals’ faces of two gender categories in an alternating condition. The neural processing engaged in gender categorization of faces was identified by assessing RS of brain activities in response to faces in the repetition (vs. alternating) condition. The analysis of event-related potentials (ERPs) to faces found reliable gender-related RS of a positive activity around 200 ms after face onset at the frontal-central region (i.e. the P2 component) in the repetition (vs. alternating) condition (Zhang et al. 2023). This finding uncovered a neural process that is similar for faces of the same gender and provides a neural index of gender categorization of faces. In addition, the gender-related RS effect on the P2 amplitudes was observed in a one-back task that required participants to respond to the immediate repetition of the same face in two consecutive trials. The one-back task forced participants to explicitly focus on how an individual face is different from any other faces (i.e. individuation) and thus did not allow participants to explicitly examine how an individual face is similar to other faces in gender (i.e. gender categorization). Because individuation and categorization are two competitive processes and enhanced individuation of faces occurs with decreased categorization of faces (Hugenberg et al. 2010, Han 2018), the one-back task prompted the processing of individuation but not categorization. In this sense, gender categorization of faces as indexed by the reliable RS effects on the P2 amplitudes occurred spontaneously. Therefore, the RS paradigm allowed us to test whether prioritization of gender categorization of same-sex faces would occur spontaneously.

However, the results reported in the previous work combined ERPs to female and male faces in women and men (Zhang et al. 2023). In the present study, we analysed ERPs to female and male faces in the RS paradigm, respectively. We compared the RS effects of neural responses to female and male faces in two gender samples. The perception bias hypothesis predicted stronger RS of the P2 amplitudes to female than male faces in both women and men. The social task demand hypothesis, however, predicted stronger RS of the P2 amplitudes to female (vs. male) faces in women but to male (vs. female) faces in men. Our results provide evidence for the latter prediction and contribute to our understanding of the cognitive and neural processes supporting gender categorization of faces in human adults.

## Methods

### Participants

The sample sizes were estimated using MorePower 6.0.4 (Campbell and Thompson 2012) based on the effect size of a two-way interaction on RS of the P2 amplitudes related to gender categorization (Zhang et al. 2023, Study 2, *ƞ_p_^2^ *= 0.173). Forty-six participants were required to detect a significant Condition (alternating vs. repetition) × Face Sex (male vs. female) interaction (α = 0.05, power = 0.85). To allow for dropout due to EEG recording failure, we aimed to recruit at least 50 participants for each gender sample. Fifty-three female and 63 male Chinese students were initially recruited. Participants’ sexual orientation were assessed using the Erotic Response and Orientation Scale (Storms 1980). Three female and one male participants were excluded due to their homosexual orientations, leaving 50 female (mean age = 21.22 ± 2.65 years) and 62 male participants (average age = 21.26 ± 2.53 years) for data analyses. All participants were right handed, had normal or corrected-to-normal vision, and reported no neurological or psychiatric history. All participants provided written informed consent after the experimental procedure had been fully explained. This study was approved by a local research ethics committee.

### Stimuli and procedures

Sixteen male and 16 female face images with neutral expressions were adopted from the previous studies (Sheng and Han 2012, Wang and Han 2021). Luminance levels were adjusted and matched between female and male faces. Each stimulus with 200 × 250 pixels subtended a visual angle of 4.9°× 6.1° at a viewing distance of 65 cm.

During EEG recording, each trial consisted of a face that was displayed for 200 ms at the centre of a grey background followed by a fixation cross with a duration varying randomly between 250 and 550 ms. Participants were instructed to perform a one-back task by pressing a button to respond to the immediate repetition of the same face in two consecutive trials. Participants were encouraged to respond as fast and accurately as possible. Each EEG recording session consisted of two runs. There were four blocks in the alternating condition and four blocks in the repetition condition (two blocks for female faces and two blocks for male faces) in each run. In the repetition condition, faces of the same sex were presented in a random order within each block. In the alternating condition, female and male faces were displayed in a random order within each block. Each block contained 18 trials, including 16 nontarget faces and 2 target faces. The blocks in the repetition or alternating condition in each run were presented in a random order. There was an 8-s break between two consecutive blocks during which a countdown from 8 to 1 appeared at the fixation position with each number flashing for 1 s. This design ensured an equal number (i.e. 128) of nontarget trials for each gender category in both the alternating and repetition conditions.

### EEG data acquisition and analysis

EEG data were continuously recorded from 64 Ag/AgCl ring electrodes mounted in a 10–20 system cap, with an additional electrode below the right eye. The EEG signals were collected at a sampling rate of 500 Hz (BrainAmp DC; Brain Products GmbH, Gilching, Germany), referenced online against FCz. The electrode AFz was used as the ground. Electrode impedance was maintained at less than 5 kΩ. During offline processing, the EEG data were re-referenced to the left and right mastoid electrodes (TP9, TP10) and filtered with a 1–30 Hz band-pass filter. ERPs in each condition were averaged separately offline, with an epoch beginning 200 ms before stimulus onset and continuing for 600 ms. The baseline for ERP amplitude measurements was the mean voltage of a 200 ms prestimulus interval. All ERP data were baseline corrected. EEG epochs contaminated by blinks or head movements were automatically removed using the independent component analysis procedure. Trials contaminated by eye movement, eye blinks, and muscle potentials exceeding ±100 μV at any electrode were excluded from the average. Only EEG signals from nontarget faces (128 trials in each condition) were included for analyses. The artefact rejection process excluded 0.78% of trials from further EEG data analyses.

The mean amplitudes of the P2 (170–210 ms) were calculated from the frontal-central electrodes (Fz, F3, F4, FCz, FC3, FC4, Cz, C3, C4). The mean amplitudes of the P270 (240–330 ms) derived were calculated from the left (PO7, P5, P7) and right (PO8, P6, P8) occipital-temporal electrodes, respectively. Both behavioural performances and ERP amplitudes were subject to repeated-measure analyses of variance (ANOVAs) with Condition (alternating vs. repetition) and Face Sex (female vs. male) as within-subjects variables and Observers’ Gender (women vs. men) as a between-subjects variable.

## Results

Response accuracies in the one-back task during EEG recording were high (>88%). ANOVAs of reaction times and response accuracies did not show any significant effect (*P*s > .07), suggesting comparable task difficulty and attentional demand in target detection in different conditions and gender samples.

**Figure. 1.**
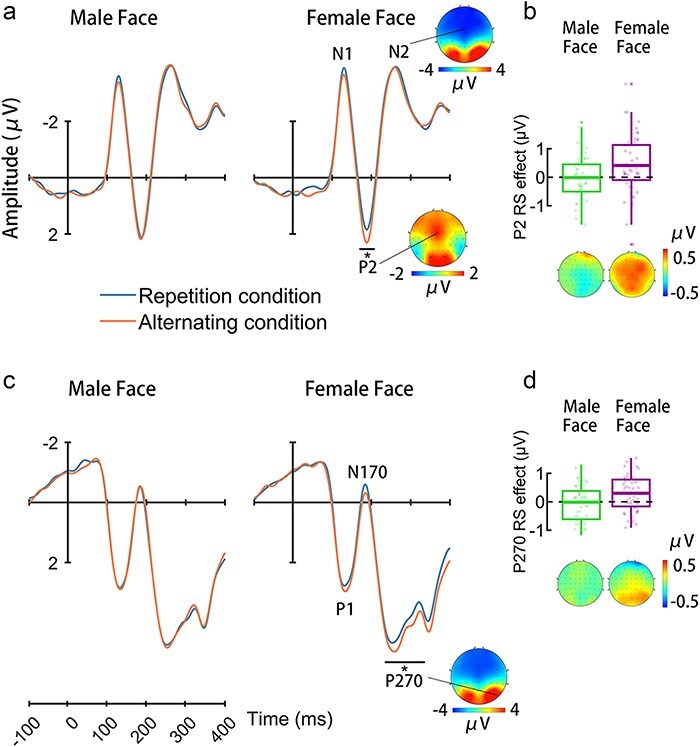
ERP results in women. (a) ERPs to male and female faces at the frontal-central electrodes. The voltage topography illustrates the scalp distributions for the P2 amplitude. (b) The mean RS effect on the P2 amplitude to female and male faces. The topographies show the scalp distributions for the P2 RS effect. (C) ERPs to male and female faces at the right occipital-temporal electrodes. The voltage topography illustrates the scalp distributions for the P270 amplitude. (d) The mean RS effect on the P270 amplitude to female and male faces. The topographies show the scalp distributions for the P270 RS effect. Shown in (c) and (d) are the average RS values (the bars in the boxes), interquartile range (the boxes), and individual participant data (small dots). The whiskers extend to the furthest nonoutlier data points, while outliers, marked as asterisks (*), are those beyond 1.5 times of the interquartile range from the box edges.

**Figure. 2.**
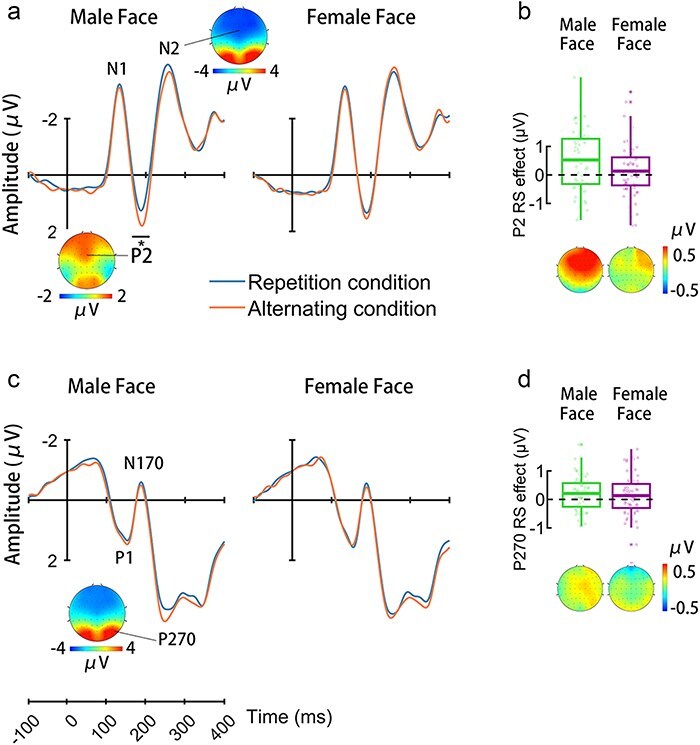
ERP results in men. (a) ERPs to male and female faces at the frontal-central electrodes. The voltage topography illustrates the scalp distributions for the P2 amplitude. (b) The mean RS effect on the P2 amplitude to female and male faces. The topographies show the scalp distributions for the P2 RS effect. (c) ERPs to male and female faces at the right occipital-temporal electrodes. The voltage topography illustrates the scalp distributions for the P270 amplitude. (d) The mean RS effect on the P270 amplitude to female and male faces. The topographies show the scalp distributions for the P270 RS effect. Shown in (c) and (d) are the average RS values (the bars in the boxes), interquartile range (the boxes), and individual participant data (small dots). The whiskers extend to the furthest nonoutlier data points, while outliers, marked as asterisks (*), are those beyond 1.5 times of the interquartile range from the box edges.

ERPs to nontarget faces were characterized by an early negative wave peaking at 130 ms (N1) and a positive wave at 170–210 ms (P2), which were followed by a negative wave at 260 ms (N2) over the frontal-central electrodes (Figs 1a and 2a). Face stimuli also elicited a positive activity at 144 ms (P1), a negativity at 186 ms (N170), and a long-latency positive deflection peaking at 270 ms (P270 at 240–330 ms) at the lateral occipital-temporal electrodes (Figs 1c and 2c). Based on pervious ERP results (Zhang et al. 2023), the present study focused on statistical analyses of the P2 and P270 amplitudes.

ANOVAs of the mean P2 amplitudes showed a significant three-way interaction of Condition × Face Sex × Observers’ Gender (F(1110) = 8.436, *P* = .004, *ƞ_p_^2^ *= 0.071, 90% CI = [0.013, 0.157]), suggesting distinct patterns of RS of the P2 amplitudes in women and men. Separate analyses of the P2 amplitudes revealed significant Condition × Face Sex interactions in both women (F(1,49) = 4.321, *P* = .043, *ƞ_p_^2^ *= 0.081, 90% CI = [0.001, 0.216]) and men (F(1,61) = 4.200, *P* = .045, *ƞ_p_^2^ *= 0.064, 90% CI = [0.001, 0.180]). Further simple effect analyses of the P2 amplitudes in women identified a significant RS effect on the P2 amplitudes to female faces (F(1,49) = 7.921, *P* = .007, *ƞ_p_^2^ *= 0.139, 90% CI = [0.023, 0.285]) but not to male faces (F(1,49) = 0.010, *P* = .922, *ƞ_p_^2^ *= 0.000, 90% CI = [0.000, 0.008]). To further assess the null RS effect on the P2 amplitudes in response to male faces, we conducted Bayes factor analyses for repeated measures ANOVA. The results support the null hypothesis (Bayes factor ${\mathrm{B}}{{\mathrm{F}}_{10}}$=0.15). In contrast, simple effect analyses in men found a significant RS effect on the P2 amplitudes in response to male faces (F(1,61) = 14.980, *P* < .001, *ƞ_p_^2^ *= 0.197, 90% CI = [0.066, 0.332]), not to female faces (F(1,61) = 1.306, *P* = .258, *ƞ_p_^2^ *= 0.021, 90% CI = [0.000, 0.110], Bayes factor ${\mathrm{B}}{{\mathrm{F}}_{10}}$=0.26). These results provide evidence for RS of an early process shared by faces that are of the same sex with observers.

Similarly, ANOVAs of the P270 amplitudes at the right occipital-temporal electrodes revealed a significant Condition × Face Sex × Observers’ Gender interaction (F(1110) = 7.322, *P* = .008, *ƞ_p_^2^ *= 0.062, 90% CI = [0.009, 0.146]). Separate analyses of the P270 amplitudes revealed a significant Condition × Face Sex interaction in women (F(1,49) = 5.544, *P* = .023, *ƞ_p_^2^ *= 0.102, 90% CI = [0.008, 0.242]) but not in men (F(1,61) = 0.560, *P* = .457, *ƞ_p_^2^ *= 0.009, 90% CI = [0.000, 0.083], Bayes factor ${\mathrm{B}}{{\mathrm{F}}_{10}}$=0.22). Further simple effect analyses showed a significant RS effect on the P270 amplitudes to female faces (F(1,49) = 10.921, *P* = .002, *ƞ_p_^2^ *= 0.182, 90% CI = [0.045, 0.331]) but not to male faces (F(1,49) = 0.027, *P* = .870, *ƞ_p_^2^ *= 0.001, 90% CI = [0.000, 0.022], Bayes factor ${\mathrm{B}}{{\mathrm{F}}_{10}}$=0.16) in women. There was a significant main effect of Condition on the P270 amplitudes in men (F(1,61) = 6.501, *P* = .013, *ƞ_p_^2^ *= 0.096, 90% CI = [0.011, 0.221]). Similar analyses of the P270 amplitudes at the left occipital-temporal electrodes failed to show significant Condition  × Face Sex  × Observers’ Gender interaction (F(1110) = 0.368, *P* = .545, *ƞ_p_^2^ *= 0.003, 90% CI = [0.000, 0.043]). These results suggested a priority of female over male faces in the late neural process of gender categorization in women.

To clarify whether emotional responses to perceived faces contributed to the observed RS effects on the P2 and P270 amplitudes, after EEG recording, we recalled the participants (42 women, 57 men) to report their arousal related to each face on a 7-point scale (1 = calm, 7 = excited). If stimulus-induced arousal determined the RS effects on the P2 and P270 amplitudes, we would expect that women would report higher arousal related to female (vs. male) faces, whereas men would report higher arousal related to male (vs. female) faces. However, the result showed slightly higher arousal related to female (vs. male) faces in both women (mean ± SD = 3.2 ± 1.01 vs. 2.6 ± 0.92, t(41) = 4.805, *P* < .001, Cohen’s d = 0.741, 90% CI of the difference = [0.340, 0.832]) and men (Mean ± SD = M = 3.0 ± 0.94 vs. 2.7 ± 0.87, T(56) = 3.416, *P* = .001, Cohen’s d = 0.452, 90% CI of the difference = [0.119,0.457]), suggesting that emotional responses cannot consistently account for the RS effects on the P2 and P270 amplitudes in women and men. We also conducted correlation analyses across the participants to examine potential relationships between self-reports of arousal and the RS effects on the P2 and P270 amplitudes. However, no significant result was observed for either women (P2/P270 amplitudes: r = 0.170/0.117, *P* = .281/.462) or men (P2/P270 amplitudes: r = 0.046/0.104, *P* = .735/.441). Together, these results provided no evidence that the patterns of the RS effects on neural responses to female or male faces in women and men can be simply attributed to arousal induced by these faces.

## Discussion

We recorded EEG signals to faces in a RS paradigm to test distinct predictions derived from the perception bias hypothesis (Gandolfo and Downing 2020) and the social task demand hypothesis (Zhang et al. 2023) regarding the prioritization of gender categorization of same-sex faces or different-sex faces in women and men. We found that the P2 responses over the frontal-central regions were decreased when male or female faces were displayed in the repetition than alternating conditions. This result replicated the previous findings (Zhang et al. 2023) and provide further evidence for early neural processes involved in coding sex similarity of faces during gender categorization of faces. Importantly, we found evidence for distinct patterns of the P2 RS effects on female and male faces in women and men. These findings help to clarify the different predictions derived from the two hypotheses.

Specifically, we found evidence that women showed reliable RS of the P2 responses to female but not to male faces. For men, however, RS occurred to the P2 responses to male faces but not to female faces. Our findings indicate prioritization of gender categorization of female over male faces in women but a reverse pattern in men. These ERP results do not fit the prediction of the perception bias hypothesis (Gandolfo and Downing 2020) but are consistent with the prediction of the social task demand hypothesis (Zhang et al. 2023). The prioritization of gender categorization of same-sex faces is important for fast classification of mating competitors during mating partner selection. In contrast, to find an appropriate different-sex individual for a mating partner requires individuation of different-sex faces, which may account for the absence of early categorical neural representations of different-sex faces in both women and men. While representations of information about human faces in young infants may be affected by the gender of their caregivers (Quinn et al. 2002), social task demands and life experiences during adulthood may shape an accustomed pattern of cognitive processes (in the sense of temporal order of gender categorization of same-sex and different-sex faces) involved in gender categorization of faces.

The results of our correlation analyses suggest that the RS effects on the P2 amplitudes were not associated with arousal induced by the face stimuli in both women and men. Therefore, the differential RS effects on the P2 amplitudes in response to same-sex and different-sex faces cannot be attributed to distinct emotional responses to female and male faces in the two gender samples. Instead, observers’ own gender identifications may play a key role in the prioritization in gender categorization of same-sex faces.

Our ERP findings have valuable implications for understanding the cognitive mechanisms of social categorization of faces. The previous ERP studies employing the same paradigm found that the P2 RS effect related to spontaneous racial categorization of faces took place in response to White (but not Asian) faces in Chinese participants but to Asian (but not White) faces in White participants (Zhou et al. 2020). In addition, the P2 RS effect related to spontaneous gender categorization of faces occurred to Asian (but not White) faces in Chinese participants but to White (but not Asian) faces in White participants (Zhang et al. 2023). Because these results were obtained when Chinese and White participants viewed the same set of face stimuli, the findings indicate that racial relationships between observers and perceived targets but not perceptual features of perceived faces determined the priority of racial/gender categorization of faces of a specific social group. Similarly, because the same set of face stimuli were presented to the two gender samples in our work, our findings highlighted the critical role of gender relationships between observers and perceived targets in determining the priority of gender categorization of female or male faces. These findings together demonstrate that the priority of social categorization of others is dominated by top-down processes required by the social task demands such as dealing with race-based ingroup/outgroup or reacting to mating competitors/targets in different manners.

We also found evidence for the priority of late processes involved in gender categorization of same-sex over different-sex faces. Women showed a significant RS effect on the occipital-temporal P270 amplitudes to female (but not to male) faces, suggesting two subsequent categorical representations of same-sex faces in women that shifted from the frontal-central regions to the posterior occipital-temporal regions. It is likely that, after the early categorical representations of female faces, women may engage further processes of perceptual features shared by same-sex faces. Because men showed similar RS effect on the occipital-temporal P270 amplitudes to female and male faces, future work should test whether men have a similar selective cognitive operation during gender categorization of male faces.

Previous ERP research on gender-related face processing reported distinct neural responses to female and male faces (e.g. P2 and P3, Ito and Urland 2003, Miller et al. 2020). Our work focused on neural processes of similarity of same-sex faces raise a few open issues for future research. For example, our ERP results unraveled the temporal priority of gender categorization of same-sex faces but did not identify the relevant neural origins. Previous functional magnetic resonance imaging (fMRI) research examined RS of neural responses to two successive same-sex (vs. different-sex) faces in the occipital and temporal cortices but did not analyse the RS effects of neural responses to male and female faces and in women and men separately (Podrebarac et al. 2013). Future research can integrate fMRI or magnetoencephalography with our design to identify the neural circuits involved in gender categorization of same-sex faces in women and men, respectively.

Finally, a few limitations of the current work should be highlighted. For example, our ERP results unraveled an accustomed pattern of cognitive processes involved in spontaneous gender categorization of faces which is consistent with the social task demand hypothesis. However, we did not test the direct effect of a specific social task on gender categorization of faces. This should be examined in future work by recording neural activities underlying spontaneous gender categorization of faces and manipulating social task demands. While the one-back task employed in our study did not bias attention to either female or male faces, the current study did not test the neural RS effects related to gender categorization of faces under different social task demands. For example, it is unclear whether and how spontaneous gender categorization of different-sex faces is influenced by tasks that require judgments on facial expressions. It is also an open issue whether different cognitive strategies are employed between women and men when they are asked to explicitly classify faces by gender. In addition, although we found prioritization of spontaneous gender categorization of same-sex faces in young adults, it remains unclear whether similar cognitive strategy is employed in spontaneous gender categorization of faces in other age groups. Finally, our work did not consider the effect of gender stereotype in numerous domains (Ellemers 2018) on gender categorization of faces. These issues should be addressed in future work.

## Data Availability

The data and the codes used to generate the figures in this study are openly available to the public at https://osf.io/xem75/?view_only=8818549720bc4fc39c6f78f727b3a005.
